# Foot Pose Estimation Using an Inertial Sensor Unit and Two Distance Sensors

**DOI:** 10.3390/s150715888

**Published:** 2015-07-03

**Authors:** Pham Duy Duong, Young Soo Suh

**Affiliations:** Department of Electrical Engineering, University of Ulsan, Namgu, Ulsan 680-749, Korea; E-Mail: duyduongd2@gmail.com

**Keywords:** inertial sensor, distance sensor, foot pose estimation, Kalman filters

## Abstract

There are many inertial sensor-based foot pose estimation algorithms. In this paper, we present a methodology to improve the accuracy of foot pose estimation using two low-cost distance sensors (VL6180) in addition to an inertial sensor unit. The distance sensor is a time-of-flight range finder and can measure distance up to 20 cm. A Kalman filter with 21 states is proposed to estimate both the calibration parameter (relative pose of distance sensors with respect to the inertial sensor unit) and foot pose. Once the calibration parameter is obtained, a Kalman filter with nine states can be used to estimate foot pose. Through four activities (walking, dancing step, ball kicking, jumping), it is shown that the proposed algorithm significantly improves the vertical position estimation.

## 1. Introduction

Foot pose (position and orientation) estimation is used in many areas, such as gait analysis [1–3], exergaming [4,5] and pedestrian navigation systems [6]. The most accurate method to estimate foot pose is using an optical motion tracker. However, optical motion tracking is rather expensive, and it can only capture the motion in a limited space, which is determined by the number of cameras.

Recently, inertial sensor-based foot pose estimation methods are increasingly used, since the sensor can be attached to a shoe, which does not require any infrastructure in an environment and, thus, removes the space constraint imposed by optical motion trackers. As inertial sensors are becoming less expensive and smaller, inertial sensor-based motion tracking (ISBMT) is expected to be more popular.

The basic principle of an ISBMT is integration of gyroscope output (which gives the orientation) and double integration of accelerometer outputs (which gives the position). In the process, the sensors noises are also integrated. For example, if there is a nonzero accelerometer sensor bias term, it affects the position estimation error proportional to the square of elapsed time [7]. Furthermore, initial orientation error also significantly affects the position estimation error, since the gravitational acceleration is not correctly removed during the position estimation [7].

In foot pose tracking, the error increase can be mitigated using zero velocity updating [8,9]. Whenever a foot touches the ground, we know the velocity of the foot is zero. Using this zero velocity information, the estimation error can be significantly reduced [6].

Another approach to reduce the estimation error is to use a smoother [10]. The smoother uses both prior and posterior data to estimate the current state. This combination reduces the estimation error. One drawback of a smoother is that its computation cannot be done online and, thus, is not suitable for applications, such as gaming.

The pose estimation error also can be reduced by using additional sensors. In [11], a pressure sensor is attached on a shoe to accurately detect the zero velocity intervals, which increases the estimation accuracy. In [12], an ultrasound range sensor is used to measure the distance to walls. The ultrasound sensor does not improve foot pose estimation, but improves the location accuracy for indoor pedestrian navigation. In [13], a camera is used to read markers on the floor, which gives the absolute position and orientation of a foot. One disadvantage of this approach is that the markers must be installed on the floor, so the experiment space is limited by the number of makers. In [14], a camera and infrared LEDs are used to measure the relative pose between two feet.

In this paper, we present a methodology to improve the accuracy of foot pose estimation by attaching low-cost distance sensors on a shoe in addition to an inertial sensor unit. Since the distance sensor gives height information, it can help to improve height estimation accuracy. The improved accuracy could be helpful in gait analysis and exergaming applications. The foot pose estimation algorithm is implemented using a Kalman filter. To combine inertial sensors and distance sensors, the relative pose between two sensors should be known. This pose parameter is included in the proposed Kalman filter.

The paper is organized as follows. In Section 2, the system hardware and coordinate systems are introduced. In Section 3, dynamic equations of the Kalman filter for ISBMT are given. In Section 4, measurement equations of the Kalman filter are provided. In Section 5, the proposed algorithm is tested, and the estimated positions are compared with optical tracker measurement values. The discussion is given in Section 6.

## 2. System Overview

**Figure 1:**
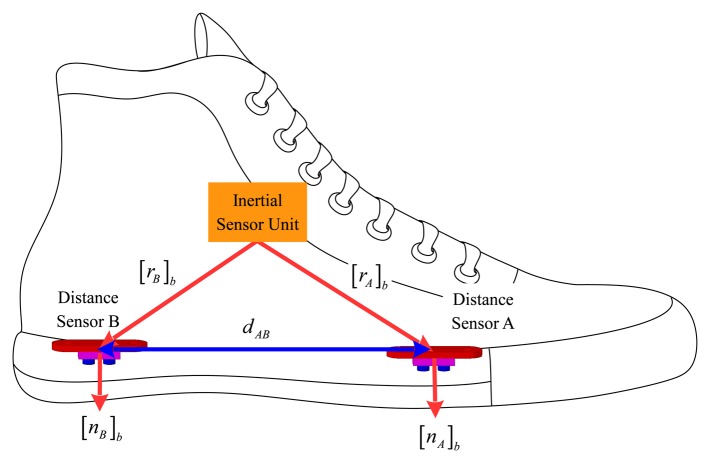
Inertial and distance sensors on a shoe.

An inertial measurement unit (three-axis accelerometers and three-axis gyroscopes, Xsens MTi) is attached on a shoe as in Figure 1. The sampling frequency of the inertial sensors is 100 Hz. Two distance sensors (VL6180 [15]) are also attached on a shoe, where symbols A and B are used to distinguish the two sensors. The distance sensor VL6180 measures the distance by measuring the time-of-flight of infrared light, and the measurement range is up to 20 cm. This sensor is most often used as a proximity sensor in smartphones. The sampling frequency of the distance sensors is 33.33 Hz.

Two coordinate systems are used in this paper: the body and world coordinate systems. The three axes of the body coordinate system coincide with the three axes of the inertial sensor unit. The *z* axis of the world coordinate system is in the direction of the local gravitational field: the *z* axis is pointing upward. The *x* and *y* axes are chosen arbitrarily. The origin of the world coordinate system is assumed to be on the floor.

The relative position and orientation of a distance sensor with respect to the inertial sensor unit are required in the foot pose estimation algorithm. As shown in Figure 1, the positions of two distance sensors are denoted [*r_A_*]*_b_* ∈ *R*^3^ and [*r_B_*]*_b_* ∈ *R*^3^, which are the position coordinates in the body coordinate system. The notation [*p*]*_b_* ([*p*]*_w_*) for a vector *p* ∈ *R*^3^ is used to emphasize that a vector is represented in the body (world) coordinate system. When there is no confusion, [*p*]*_b_* (or [*p*]*_w_*) is just expressed by *p*. The pointing direction of the distance sensor is denoted by a unit vector *n_A_* ∈ *R*^3^ and *n_B_* ∈ *R*^3^.

The distance sensor parameters (*r_A_*, *r_B_*, *n_A_*, *n_B_* in Figure 1) could be determined using a ruler and a protractor. It would be, however, not easy to determine the parameters with high accuracy. Thus, the small errors in the parameters are estimated in the Kalman filter. We model the parameters as follows:

(1) $$\begin{array}{ccc} r_{A} & = & {\hat{r}}_{A}+{\bar{r}}_{A}\\ r_{B} & = & {\hat{r}}_{B}+{\bar{r}}_{B}\\ n_{A} & = & {\hat{n}}_{A}+{\bar{n}}_{A}\\ n_{B} & = & {\hat{n}}_{B}+{\bar{n}}_{B} \end{array}$$

where *r̂_A_* ∼ *n̂_B_* are initial estimated parameter values (most likely measured values using a ruler and a protractor) and *r̄_A_* ∼ *n̄_B_* are errors in the parameter estimation.

The distance between two distance sensors is denoted by *d_AB_* = ‖*r_A_* − *r_B_*‖, which is measured by a ruler. This scalar quantity can be measured more accurately comparing with other vector parameters, which are rather difficult to measure and sometimes require guesswork. Thus, the error of *d_AB_* is not estimated. The measured value of *d_AB_* is denoted by *z_AB_*:

(2) $$z_{AB}=d_{AB}+v_{AB}$$

where *v_AB_* is the measurement noise.

## 3. Kalman Filter for ISBMT

In this section, the foot pose tracking algorithm is given.

Let *r* ∈ *R*^3^ and *v* ∈ *R*^3^ denote the position and velocity of the inertial sensor unit in the world coordinate system. Let *q* ∈ *R*^4^ be the quaternion [16] representing the rotation relationship between the body and world coordinate systems. Let *C*(*q*) ∈ *SO*(3) be the rotation matrix corresponding to the quaternion *q*.

### 3.1. Basic Pose Equations

The basic equations for *r*, *v* and *q* are given by [17]:

(3) $$\begin{array}{ccc} \dot{q} & = & \frac{1}{2}\Omega \left(\omega \right)q\\ \dot{v} & = & C{\left(q\right)}^{\prime }{\left[a\right]}_{b}\\ \dot{r} & = & v \end{array}$$

where [*a*]*_b_* ∈ *R*^3^ is the acceleration expressed in the body coordinate system and *ω* = [*ω_x_ ω_y_ ω_z_*]′ is the angular velocity of the body coordinate system with respect to the world coordinate system. The symbol *Ω*(*ω*) is defined by:

$$\Omega \left(\omega \right)≜\left[\begin{array}{cccc} 0 & -\omega_{x} & -\omega_{y} & -\omega_{z}\\ \omega_{x} & 0 & \omega_{z} & -\omega_{y}\\ \omega_{y} & -\omega_{z} & 0 & \omega_{x}\\ \omega_{z} & \omega_{y} & -\omega_{x} & 0 \end{array}\right]$$

### 3.2. Numerical Integration

The basic principle of ISBMT is that *q*, *v* and *r* can be computed by numerically integrating Equation (3) [7]. To do that, we need to know *ω* and [*a*]*_b_*, which can be measured using gyroscopes and accelerometers.

The gyroscope output (*y_g_* ∈ *R*^3^) and accelerometer output (*y_a_* ∈ *R*^3^) can be modeled as follows:

(4) $$\begin{array}{lll} y_{g} & = & \omega +v_{g}\\ y_{a} & = & {\left[a\right]}_{b}+C\left(q\right){\left[\tilde{g}\right]}_{w}+v_{a} \end{array}$$

where [*g̃*]*_w_* = [ 0 0 *g* ]′ ∈ *R*^3^ is the local gravitational vector in the world coordinate system and *g* is the magnitude of the gravitational acceleration. The measurement noises *v_g_* and *v_a_* are assumed to be uncorrelated zero mean white Gaussian.

We can integrate Equation (3) by replacing *ω* by *y_g_* and replacing [*a*]*_b_* by *y_a_ − C*(*q*)*g̃*. The numerical integration algorithm is given in [18]. Let the integrated values be denoted by *q̂, r̂* and *v̂*.

Since *ω* ≠ *y_g_* and [*a*]*_b_* ≠ *y_a_* − *C*(*q̂*)*g̃*, there are errors in *q̂*, *r̂* and *v̂*, which are denoted by *q̄* ∈ *R*^3^, *r̄* ∈ *R*^3^ and *v̄* ∈ *R*^3^:

(5) $$\begin{array}{lll} \bar{q} & = & \left[\begin{array}{cc} 0_{3\times 1} & I_{3} \end{array}\right]\left({\hat{q}}^{*}⊗q\right)\\ \bar{r} & = & r-\hat{r}\\ \bar{v} & = & v-\hat{v} \end{array}$$

where ⊗ denotes the quaternion multiplication and q* is the conjugate quaternion of *q* ∈ *R*^4^. The definition of *q̄* ∈ *R*^3×1^ is from the assumption that the estimation error of *q̂* is small, and thus, the following is satisfied [19]:

(6) $$q\approx \hat{q}⊗\left[\begin{array}{c} 1\\ \bar{q} \end{array}\right]$$

The errors *q̄*, *r̄* and *v̄* along with the parameter errors *r̄_A_* ∼ *n̄_B_* in Equation (1) are estimated in the Kalman filter. Combining nine states in Equation (5) and 12 states in Equation (1), we have the following state for a Kalman filter:

(7) $$x=\left[\begin{array}{c} \bar{q}\\ \bar{r}\\ \bar{v}\\ {\bar{r}}_{A}\\ {\bar{r}}_{B}\\ {\bar{n}}_{A}\\ {\bar{n}}_{B} \end{array}\right]\in R^{21\times 1}$$

Once the calibration parameters *r̂_A_* ∼ *n̂_B_* are estimated, the parameter error terms *r̄_A_* ∼ *n̄_B_* in Equation (7) can be removed for fast computation. The dynamic equation of *x* is given by:

(8) $$\dot{x}\left(t\right)=A\left(t\right)x\left(t\right)+w\left(t\right)$$

where:

$$A\left(t\right)=\left[\begin{array}{ccccccc} \left[-y_{g}\times \right] & 0 & 0 & 0 & 0 & 0 & 0\\ 0 & 0 & I_{3} & 0 & 0 & 0 & 0\\ -2C{\left(\hat{q}\right)}^{\prime }\left[y_{a}\times \right] & 0 & 0 & 0 & 0 & 0 & 0\\ 0 & 0 & 0 & 0 & 0 & 0 & 0\\ 0 & 0 & 0 & 0 & 0 & 0 & 0\\ 0 & 0 & 0 & 0 & 0 & 0 & 0\\ 0 & 0 & 0 & 0 & 0 & 0 & 0 \end{array}\right],w\left(t\right)=\left[\begin{array}{c} -\frac{1}{2}v_{g}\\ 0\\ -C{\left(\hat{q}\right)}^{\prime }v_{a}\\ 0\\ 0\\ 0\\ 0 \end{array}\right]$$

The dynamic equations for *q̄*, *r̄* and *v̄* are from the result in [20]. The derivatives of *r̄_A_* ∼ *n̄_B_* are zero, since *r_A_* ∼ *n_B_* are constant parameters.

## 4. Measurement Equation of the Kalman Filter

Two measurement equations are used in the Kalman filter. One is the measurement from distance sensors (Section 4.1), and the other is the measurement equation using the zero velocity intervals (Section 4.2).

### 4.1. Distance Sensor Output and Parameters

The distance sensor outputs *z_A_* ∈ *R* and *z_B_* ∈ *R* can be modeled as follows:

(9) $$\begin{array}{ccc} z_{A} & = & d_{A}+v_{A}\\ z_{B} & = & d_{B}+v_{B} \end{array}$$

where *d_A_* and *d_B_* are true distance values of sensors A and B, respectively. Symbols *v_A_* and *v_B_* denote measurement noises of *A* and *B*, respectively. We note that *d_A_* and *d_B_* are not the same as the heights of distance sensors A and B if they are not perpendicular to the floor. The heights can be obtained from *d_A_* and *d_B_* if we know the orientation of distance sensors.

Assuming that the floor is flat and the origin of the world coordinate system lies on the floor, the following is satisfied:

(10) $$\begin{array}{ccc} \left[\begin{array}{ccc} 0 & 0 & 1 \end{array}\right]{\left[r\right]}_{w} & = & -\left[\begin{array}{ccc} 0 & 0 & 1 \end{array}\right]C{\left(q\right)}^{\prime }{\left[r_{A}+n_{A}d_{A}\right]}_{b}\\ \left[\begin{array}{ccc} 0 & 0 & 1 \end{array}\right]{\left[r\right]}_{w} & = & -\left[\begin{array}{ccc} 0 & 0 & 1 \end{array}\right]C{\left(q\right)}^{\prime }{\left[r_{B}+n_{B}d_{B}\right]}_{b} \end{array}$$

In Equation (10), − (*r_a_* + *n_a_d_a_*) is a vector (in the body coordinate system) from a point (intersection of line *n_a_* and floor plane) on the floor to the inertial sensor. By pre-multiplying *C*(*q*)′ (the rotation matrix from the body coordinate system to the world coordinate system), the third component of *C*(*q*)′ (*r_a_* + *n_a_d_a_*) is the height of the inertial sensor.

Now, we relate Equation (10) to the state Equation (7). Note that [21]:

(11) $$C{\left(q\right)}^{\prime }=C{\left(\hat{q}\right)}^{\prime }\left(I+2\left[\bar{q}\times \right]\right)$$

Inserting Equations (1), (5), (9) and (11), into the first equation of Equation (10), we have the following:

(12) $$-\left[\begin{array}{ccc} 0 & 0 & 1 \end{array}\right]C{\left(\hat{q}\right)}^{\prime }\left(I+2\left[\bar{q}\times \right]\right)\left({\hat{r}}_{A}+{\bar{r}}_{A}+\left({\hat{n}}_{A}+{\bar{n}}_{A}\right)\left(z_{A}-v_{A}\right)\right)=\left[\begin{array}{ccc} 0 & 0 & 1 \end{array}\right]\left({\hat{r}}_{A}+\bar{r}\right)$$

Assuming that the error terms (*q̄*, *r̄_A_*, *n̄_A_*, *r̄*) and the noise term (*v_a_*) are small, we can ignore the product terms: for example, [*q̄*×]*r̄_A_* is ignored. By ignoring the product terms, we obtain:

(13) $${\tilde{z}}_{A}=\left[\begin{array}{ccc} 0 & 0 & 1 \end{array}\right]\left(2C{\left(\hat{q}\right)}^{\prime }\left[\left({\hat{r}}_{A}+{\hat{n}}_{A}z_{A}\right)\times \right]\bar{q}-\bar{r}-C{\left(\hat{q}\right)}^{\prime }{\bar{r}}_{A}-C{\left(\hat{q}\right)}^{\prime }z_{A}{\bar{n}}_{A}+C{\left(\hat{q}\right)}^{\prime }{\hat{n}}_{A}v_{A}\right)$$

where:

$${\tilde{z}}_{A}=\left[\begin{array}{ccc} 0 & 0 & 1 \end{array}\right]\left(\hat{r}+C{\left(\hat{q}\right)}^{\prime }\left({\hat{r}}_{A}+{\hat{n}}_{A}z_{A}\right)\right)\in R$$

Thus, the measurement *z̃_A_* is related to the state ***x*** as follows:

(14) $${\tilde{z}}_{A}=H_{A}x+C{\left(\hat{q}\right)}^{\prime }{\hat{n}}_{A}v_{A}$$

where:

$$H_{A}=\left[\begin{array}{ccc} 0 & 0 & 1 \end{array}\right]\left[\begin{array}{ccccccc} 2C{\left(\hat{q}\right)}^{\prime }\left[\left({\hat{r}}_{A}+{\hat{n}}_{A}z_{A}\right)\times \right] & -I_{3} & 0 & -C{\left(\hat{q}\right)}^{\prime } & 0 & -C{\left(\hat{q}\right)}^{\prime }z_{A} & 0 \end{array}\right]\in R^{1\times 21}$$

Similarly, we can derive a measurement equation for the distance sensor B:

(15) $${\tilde{z}}_{B}=H_{B}x+C{\left(\hat{q}\right)}^{\prime }{\hat{n}}_{B}v_{B}$$

where:

$$\begin{array}{c} {\tilde{z}}_{B}=\left[\begin{array}{ccc} 0 & 0 & 1 \end{array}\right]\left(\hat{r}+C{\left(\hat{q}\right)}^{\prime }\left({\hat{r}}_{B}+{\hat{n}}_{B}z_{B}\right)\right)\in R\\ H_{B}=\left[\begin{array}{ccc} 0 & 0 & 1 \end{array}\right]\left[\begin{array}{ccccccc} 2C{\left(\hat{q}\right)}^{\prime }\left[\left({\hat{r}}_{B}+{\hat{n}}_{B}z_{B}\right)\times \right] & -I_{3} & 0 & 0 & -C{\left(\hat{q}\right)}^{\prime } & 0 & -C{\left(\hat{q}\right)}^{\prime }z_{B} \end{array}\right]\in R^{1\times 21} \end{array}$$

In addition to Equations (13) and (15), some constraints are added in the measurement equations. The first constraint is *n_a_* and *n_b_* should be unit vectors since they represent direction vectors. The second constraint is that ‖*r_a_ − r_b_*‖ = *d_AB_*.

The constraint (*n_a_* and *n_b_* should be unit vectors) is expressed as:

(16) $$\left\|n_{A}\right\|=\left\|{\hat{n}}_{A}+{\bar{n}}_{A}\right\|={\hat{n}}_{A}^{\prime }{\hat{n}}_{A}+2{\hat{n}}_{A}^{\prime }{\bar{n}}_{A}+{\bar{n}}_{A}^{\prime }{\bar{n}}_{A}=1$$

From $\left\|{\hat{n}}_{A}\right\|={\hat{n}}_{A}^{\prime }{\hat{n}}_{A}=1$ and ${\bar{n}}_{A}^{\prime }{\bar{n}}_{A}\approx 0$ (assuming that *n̄_A_* is small), we have the following approximation:

(17) $${\hat{n}}_{A}^{\prime }{\bar{n}}_{A}=0$$

Thus, constraints on *n_A_* and *n_B_* are imposed through the following measurement equations:

(18) $$\left[\begin{array}{c} 0\\ 0 \end{array}\right]=H_{\text{constraint}}x+v_{\text{constraint}}$$

where:

$$H_{\text{constraint}}\left[\begin{array}{ccccccc} 0_{1\times 3} & 0_{1\times 3} & 0_{1\times 3} & 0_{1\times 3} & 0_{1\times 3} & {\hat{n}}_{A}^{\prime } & 0_{1\times 3}\\ 0_{1\times 3} & 0_{1\times 3} & 0_{1\times 3} & 0_{1\times 3} & 0_{1\times 3} & 0_{1\times 3} & {\hat{n}}_{B}^{\prime } \end{array}\right]\in R^{2\times 21}$$

The noise term *v_constraint_* ∈ *R*^2×1^ is an artificial noise reflecting the fact that the constraint Equation (17) is an approximation.

Inserting Equation (2) into the constraint ‖*r_A_* − *r_B_*‖ = *d_AB_*, we have:

(19) $$z_{AB}=\left\|{\hat{r}}_{A}+{\bar{r}}_{A}-\left({\hat{r}}_{B}+{\bar{r}}_{B}\right)\right\|+v_{AB}$$

Since it has the nonlinear relationship, an extended Kalman filtering technique is used for this measurement.

(20) $${\tilde{z}}_{AB}=H_{AB}x+v_{AB}$$

where:

$$\begin{array}{c} {\tilde{z}}_{AB}=z_{AB}-\left\|{\hat{r}}_{A}-{\hat{r}}_{B}\right\|\in R\\ \begin{array}{lll} H_{AB} & = & \left[\begin{array}{ccccccc} 0_{1\times 3} & 0_{1\times 3} & 0_{1\times 3} & \frac{\partial d_{AB}}{\partial {\bar{r}}_{A}} & \frac{\partial d_{AB}}{\partial {\bar{r}}_{B}} & 0_{1\times 3} & 0_{1\times 3} \end{array}\right]\\ & = & \left[\begin{array}{ccccccc} 0_{1\times 3} & 0_{1\times 3} & 0_{1\times 3} & \frac{{\left({\hat{r}}_{A}-{\hat{r}}_{B}\right)}^{\prime }}{\left\|{\hat{r}}_{A}-{\hat{r}}_{B}\right\|} & \frac{-{\left({\hat{r}}_{A}-{\hat{r}}_{B}\right)}^{\prime }}{\left\|{\hat{r}}_{A}-{\hat{r}}_{B}\right\|} & 0_{1\times 3} & 0_{1\times 3} \end{array}\right]\in R^{1\times 21} \end{array} \end{array}$$

In summary, Equations (14), (15), (18) and (20) are used as measurement equations for the Kalman filter if distance sensor data *z_A_* and *z_B_* are available. In this case, the measurement equation is given by:

$$z=\left[\begin{array}{c} {\tilde{z}}_{A}\\ {\tilde{z}}_{B}\\ 0_{2\times 1}\\ {\tilde{z}}_{AB} \end{array}\right]=\left[\begin{array}{c} H_{A}\\ H_{B}\\ H_{\text{constraint}}\\ H_{AB} \end{array}\right]x+\left[\begin{array}{c} C{\left(\hat{q}\right)}^{\prime }{\hat{n}}_{A}v_{A}\\ C{\left(\hat{q}\right)}^{\prime }{\hat{n}}_{B}v_{B}\\ v_{\text{constraint}}\\ v_{AB} \end{array}\right]$$

### 4.2. Zero Velocity Updating

In addition to distance sensors, zero velocity updating is used in a measurement equation. If we know when a foot is not moving (for example, when a foot is on the ground), the velocity error in the ISBMT algorithm can be reset, which significantly reduces foot pose estimation errors. Since velocity sensors (such as a Doppler velocity sensor in [22]) are not used, the zero velocity intervals are detected indirectly, where many zero velocity detection algorithms have been proposed [8,23].

In this paper, we use a simple zero velocity detection algorithm. Let *Z_m_* be a set of all discrete time indices belonging to the zero velocity intervals. The discrete time *k* belongs to *Z_m_* if the following conditions are satisfied:

(21) $$\begin{array}{ll} \left\|y_{g,i}\right\|\leq B_{g}, & k-\frac{N_{g}}{2}\leq i\leq k+\frac{N_{g}}{2}\\ \left\|y_{a,i}-y_{a_{i-1}}\right\|\leq B_{a}, & k-\frac{N_{a}}{2}\leq i\leq k+\frac{N_{a}}{2} \end{array}$$

where *N_g_* and *N_a_* are even number integers.

During zero velocity intervals, we assume the following:

(22) $$v=0_{3\times 1}$$

Inserting Equation (5) into Equation (22), we have the following measurement equation in the Kalman filter.

(23) $$z_{v}=H_{v}x+v_{\text{zero}}$$

where:

$$\begin{array}{c} z_{v}=0_{3\times 1}-\hat{v}\in R^{3\times 1}\\ H_{v}=\left[\begin{array}{ccccccc} 0_{3\times 3} & 0_{3\times 3} & I_{3} & 0_{3\times 3} & 0_{3\times 3} & 0_{3\times 3} & 0_{3\times 3} \end{array}\right]\in R^{3\times 21} \end{array}$$

The noise term *v_zero_* ∈ *R*^3×1^ is an artificial noise reflecting the fact that the true velocity may not be zero (the velocity may be almost zero, but not exactly zero).

## 5. Experiments and Results

**Figure 2:**
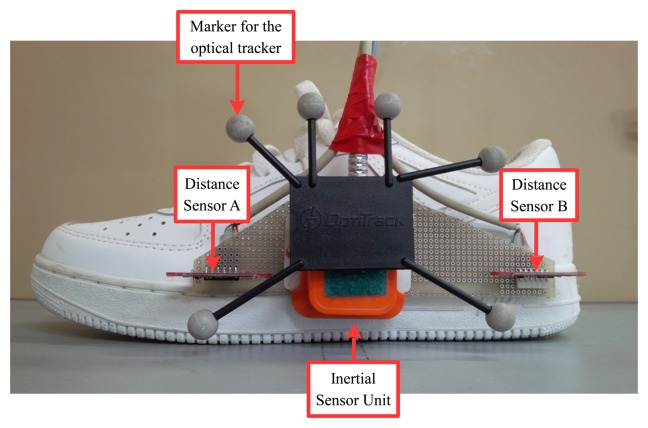
A shoe for the experiment.

The proposed algorithm is tested with several foot movement experiments. The foot position is measured with an inertial sensor unit (Xsens MTi) and two distance sensors (VL6180) as in Figure 2. The estimated positions are compared with the positions obtained using an optical motion tracker (Optitrack six Flex 13 camera system), which is considered as a ground truth.

The parameters used in the proposed algorithm are summarized in Table 1. All noises are assumed to be uncorrelated white Gaussian noises.

Four motions (walking, dancing steps, kicking in a football, jumping) are tested. The estimated position is compared with two other inertial sensor-only pose estimations. In the first inertial sensor-only pose estimation, the same algorithm is used, except for the distance sensors: that is, Equations (14) and (15) are not used in the Kalman filter. We call this method “K.F. (zero velocity updating)”. In the second inertial sensor-only pose estimation, the height updating is added in the first inertial sensor-only pose estimation. If we assume that the floor is flat, the foot height during the zero velocity intervals (that is, when a foot is on the ground) should be the same. Thus, in addition to zero velocity updating, the *z* axis value of *r* is updated to the initial *z* axis value during each zero velocity interval. We call this method “K.F. (zero velocity + height updating)”

**Figure 3:**
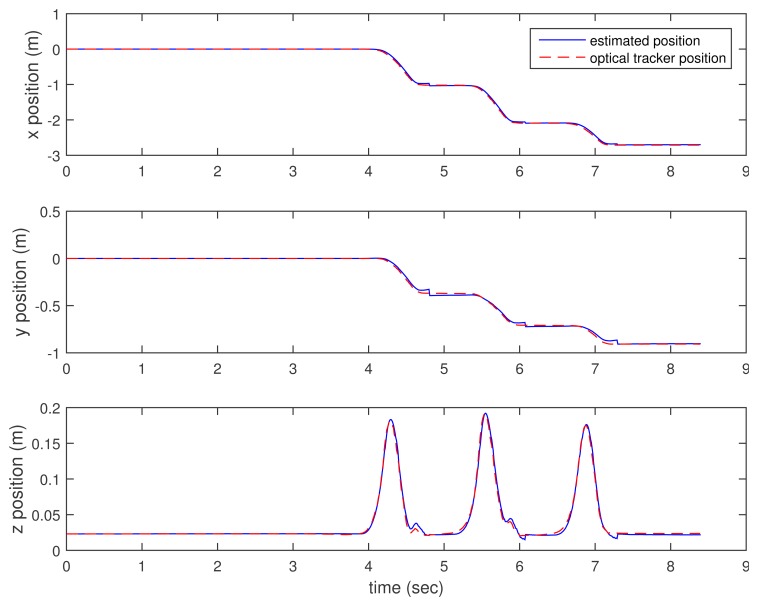
Walking position estimation: proposed method.

**Figure 4:**
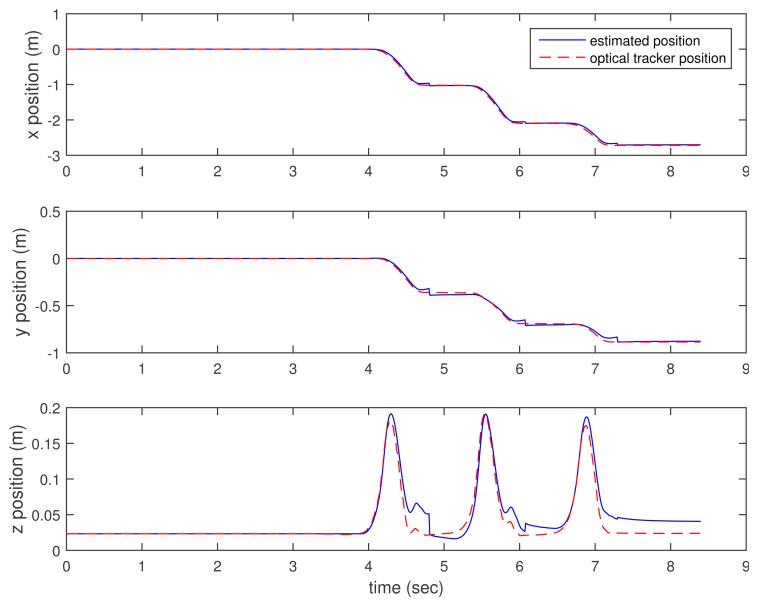
Walking position estimation: K.F. (zero velocity updating). K.F., Kalman filter.

**Figure 5:**
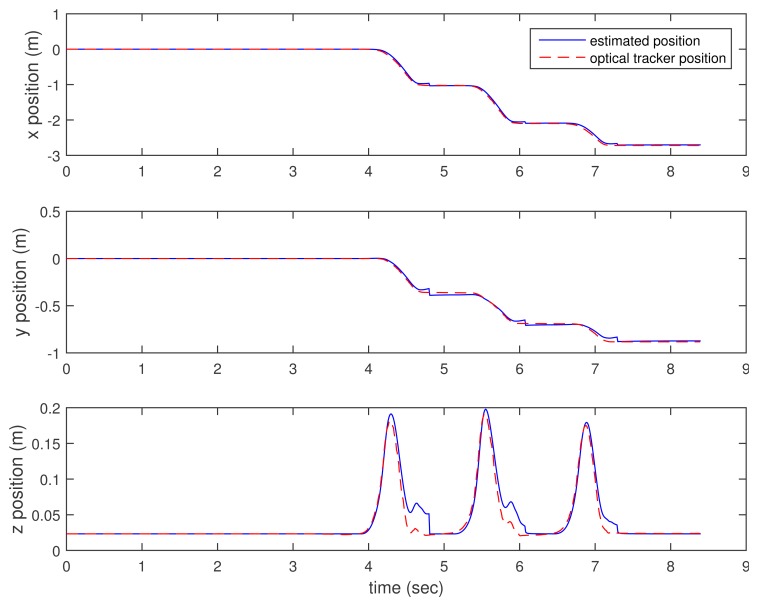
Walking position estimation: K.F. (zero velocity + height updating).

In Figures 3, 4–5, the estimated positions (walking case) by three methods (the proposed method and the two inertial sensor-only methods) are given along with the position by an optical tracker. Since the optical tracker coordinate system is different from the world coordinate system, the position data are translated and rotated. Furthermore, the inertial sensor data and optical tracker data are not synchronized at the hardware level. The data are synchronized by maximizing the cross-correlation.

We can see that *x* and *y* axis position estimations are similar in all three methods. This is not surprising, since the distance sensor only gives the *z* axis position (height) information. In the z axis position estimation, the proposed method gives better results (see the third graphs of Figures 3, 4–5 around 4.6, 6 and 7 s). Among inertial sensor-only estimations, the Kalman filter (zero velocity + height updating) is better, since its height is compensated during the zero velocity intervals.

**Figure 6:**
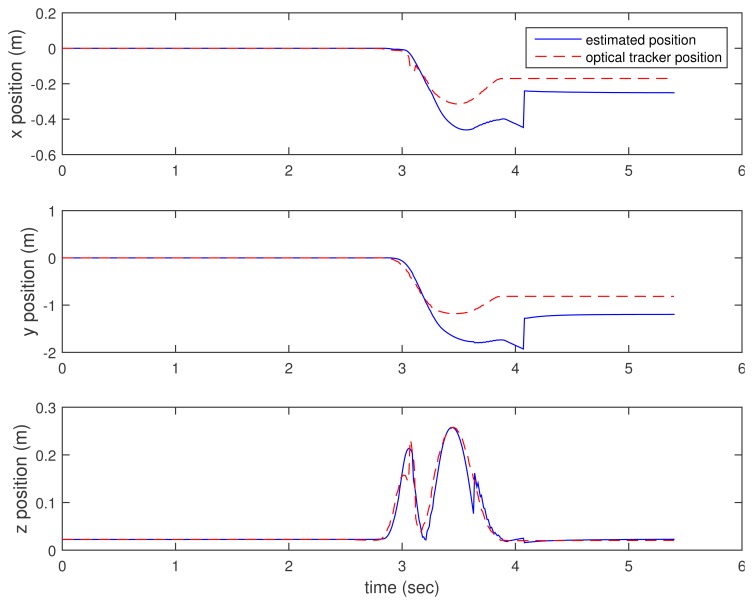
Ball kicking estimation: proposed method.

**Figure 7:**
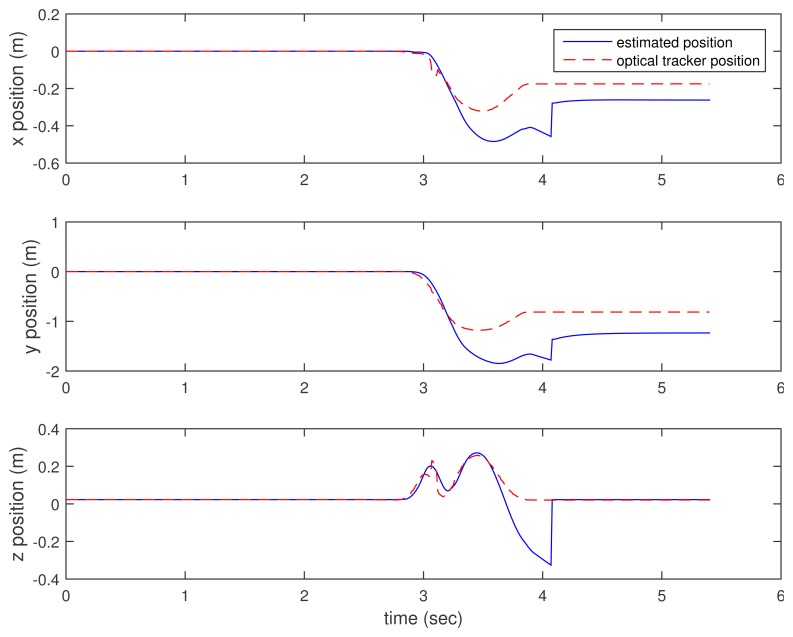
Ball kicking estimation: K.F. (zero velocity + height updating).

Another example is given in Figures 6 and 7, where a ball kicking action is done. The *x* and *y* position estimation results are similar, both by the proposed method and K.F. (zero velocity + height updating). In this case, the position errors are quite large, presumably due to the fact that there is a rather long moving interval (2.8∼4-s interval) and a quick movement (large sensor values). In the *z* axis position estimation, we can see that the proposed method gives a significantly better result.

To compare the result quantitatively, RMS (root mean square) position errors are computed. The *x* axis RMS position error is defined by:

$$E_{\text{RMS},x}=\sqrt{\frac{1}{N}\sum_{k=1}^{N}{\left({\hat{r}}_{x,k}-{\hat{r}}_{\text{optical},x,k}\right)}^{2}}$$

where *r̂_x,k_* is the *x* axis estimated position at the discrete time *k*, *r̂_optical,x,k_* is the *x* axis position measured by the optical tracker and *N* is the total number of data. Similarly, we can define *E_RMS,y_* and *E_RMS,z_*

The RMS error results for four different activities are given in Table 2. In general, there is no significant improvement in the *x* and *y* axis position estimation.

Once the calibration parameters are computed, the dimension of the Kalman filter state reduces to nine from 21, since *r̄_A_* ∼ *n̄_B_* can be removed from the state. The proposed algorithm is used with this nine-state Kalman filter. In one Kalman filter, the initial calibration parameters in Table 1 are used. In another Kalman filter, the estimated calibration parameters in Equation (24) (which are supposed to be more accurate) are used. The position estimation results of two nine-state Kalman filters are given in Table 3, where the result of the 21-state Kalman filter is repeated from Table 2 for an easy comparison. The nine-state Kalman filter with calibrated parameters gives slightly better results, except for the kicking case. Since the difference between the initial and calibrated parameters is small, the RMS error difference is also small. The calibration of parameters is a one time process, and after the calibration, we can estimate the position using the nine-state Kalman filter.

In the walking data, the calibration parameters are estimated in the Kalman filter. The calibration parameters from walking data are given by:

(24) $${\hat{r}}_{A}=\left[\begin{array}{c} -0.0124\\ 0.0806\\ 0.000 \end{array}\right],{\hat{r}}_{B}=\left[\begin{array}{c} -0.0033\\ -0.0611\\ -0.0006 \end{array}\right],{\hat{n}}_{A}=\left[\begin{array}{c} 1.0000\\ 0.0001\\ 0.0000 \end{array}\right],{\hat{n}}_{B}=\left[\begin{array}{c} 1.0000\\ 0.0002\\ -0.0001 \end{array}\right]$$

## 6. Conclusions

There are many inertial sensor-based foot pose estimation algorithms. In this paper, the position estimation is improved additionally using distance sensors. The distance sensor has a 20-cm range limitation. However, this limitation does not degrade estimation accuracy much, since a foot is over the 20-cm range only for a short time, unless the foot is in the air intentionally. Longer range sensors exist, but they are either less accurate in short ranges or more expensive.

The inertial motion estimation algorithm is proposed using a 21-state Kalman (nine states for pose tracking and 12 states for calibration parameters). After the parameters are calibrated, the nine-state Kalman filter can be used to estimate the foot pose.

The proposed algorithm is tested for four activities: walking, dancing steps, ball kicking and jumping. It is shown in Table 2 that there is significant improvement in the *z* axis position estimation compared with inertial sensor-only foot pose estimation. On the other hand, there is no improvement in the *x* and *y* axis, since the distance sensor only gives the height information.

The proposed algorithm can be used in gait analysis (which requires a foot pose estimation), motion-based gaming (such as a soccer game) and exergaming (game-based exercises).

## Figures and Tables

**Table 1:** Parameters in the proposed algorithm.

**Parameter**	**Value**	**Related Equations**
initial value of *r̂_A_*	[ −0.008 0.080 0 ]′	(1)
initial value of *r̂_B_*	[ −0.006 −0.062 0 ]′	(1)
initial value of *n̂_A_*	[1 0 0 ]′	(1)
initial value of *n̂_B_*	[1 0 0 ]′	(1)
*z_AB_*	0.142	(2)
$E\left\{v_{g}v_{g}^{\prime }\right\}$	0.00017*I*_3_	(5)
$E\left\{v_{a}v_{a}^{\prime }\right\}$	0.0083*I*_3_	(5)
*P_init_* = initial value of E{*xx′*}	*P_init_*(2 : 3, 2 : 3) = 0.001 *I*_2_(*), *P_init_*(6, 6) = 0.0001,*P_init_*(10 : 21, 10 : 21) = 0.000001*I*_12_(all unspecified elements are zero)	(7)
$E\left\{v_{A}v_{A}^{\prime }\right\}$	0.0004	(9)
$E\left\{v_{B}v_{B}^{\prime }\right\}$	0.0004	(9)
$E\left\{v_{\text{constraint}}v_{\text{constraint}}^{\prime }\right\}$	0.00000001*I*_2_	(18)
$E\left\{v_{\text{AB}}^{\prime }\right\}$	0.000001	(20)
$E\left\{v_{\text{zero}}v_{\text{zero}}^{\prime }\right\}$	0.0001*I*_3_	(23)

* *P_init_*(2 : 3, 2 : 3) represents a *R*^2×2^ matrix consisting of second and third rows and column elements.

**Table 2:** RMS position error comparison of different methods (unit: m).

**Motion Type**	**Estimation Method**	***ERMS,x***	***ERMS,y***	***ERMS,z***	**Sum**
walking	proposed method	0.0208	0.0118	0.0030	0.0356
	K.F. (zero velocity)	0.0243	0.0131	0.0121	0.0495
	K.F. (zero velocity + height updating)	0.0247	0.0130	0.0095	0.0472

dancing steps	proposed method	0.0231	0.0798	0.0084	0.1113
	K.F. (zero velocity)	0.0265	0.0805	0.0397	0.1466
	K.F. (zero velocity + height updating)	0.0269	0.0810	0.0266	0.1345

ball kicking	proposed method	0.0816	0.3513	0.0120	0.4449
	K.F. (zero velocity)	0.0871	0.3551	0.0714	0.5136
	K.F. (zero velocity + height updating)	0.0877	0.3583	0.0685	0.5145

jumping	proposed method	0.0529	0.0705	0.0188	0.1423
	K.F. (zero velocity)	0.0630	0.0807	0.0313	0.1750
	K.F. (zero velocity + height updating)	0.0637	0.0808	0.0244	0.1689

**Table 3:** RMS position error comparison of different calibration parameters (unit: m).

**Motion Type**	**Estimation Method**	***ERMS,x***	***ERMS,y***	***ERMS,z***	**Sum**
walking	proposed method	0.0208	0.0118	0.0030	0.0356
	with fixed initial parameter	0.0244	0.0119	0.0034	0.0397
	with fixed estimated parameter	0.0201	0.0119	0.0029	0.0350

dancing steps	proposed method	0.0231	0.0798	0.0084	0.1113
	with fixed initial parameter	0.0362	0.0852	0.0089	0.1303
	with fixed estimated parameter	0.0196	0.0745	0.0083	0.1024

ball kicking	proposed method	0.0816	0.3513	0.0120	0.4449
	with fixed initial parameter	0.0764	0.3495	0.0120	0.4380
	with fixed estimated parameter	0.0853	0.3506	0.0120	0.4479

jumping	proposed method	0.0529	0.0705	0.0188	0.1423
	with fixed initial parameter	0.0630	0.0807	0.0313	0.1750
	with fixed estimated parameter	0.0486	0.0655	0.0188	0.1329
